# Surgery and the General Practitioner

**Published:** 1908-02-22

**Authors:** 


### SURGERY AND THE GENERAL PRACTITIONER.

The average general practitioner is chary of attempting operations, even where he is confident of his ability to tackle the case, for various reasons. In the majority of cases, where his better class of patients are concerned, he dreads the risk of failure and the consequent loss of prestige it would entail and prefers to send the case to the specialist. He recognises, perhaps justly, that a failure means that he will gain all the blame, while success will scarcely counterbalance the risk he runs. With his poorer patients he is even less anxious to try his hand. The multiplication of general hospitals, where his patients can be treated by experts without any inconvenience or trouble to himself, are always at hand, and when a surgical case crops up in his practice he is content to send it to out-patients at the nearest clinic. The disadvantages of acting merely as a bureau of reference—a forwarding station, as it were, for the expert surgeon, gynæcologist, aurist and oculist—he has scarcely troubled himself actively to consider. Yet it is one of the most important questions which the general practitioner has to weigh. Knowledge not added to and put into practice is dead weight, and the policy of the reference bureau, not only takes work from the practitioner, but also "causes him to lose that self-confidence which is one of the most important elements in any physician's make-up." It may happen to any man in any practice to do, at some period or other of his career, a life-saving operation. When that emergency occurs no practitioner will be found wanting in courage or in confidence to shoulder the responsibility, and give his patient the only chance of life by energetically assuming the rôle of the surgeon. It is on such an occasion that the practitioner will regret his wasted opportunities, his confiding reliance on the expert, and his want of courage in tackling minor and less serious cases, from which he would have learned so much. Minor operations, if they do nothing else, will at least give the practitioner confidence in himself, a realisation of the prime value of attention to detail, of scrupulous care in asepsis, and of the necessity for careful and thoughtful consideration of all the aspects of the case. The public has the right to expect from every practitioner the knowledge and ability to diagnose and treat an urgent surgical complication, and a few of us will deny the addendum to this statement to the effect that "the education of the general practitioner at the present time is adequate to warrant him in assuming the responsibilities of grave surgical conditions." For all that the neglect of frequently offered opportunity has not put the average practitioner in a better state to cope with emergencies. What minor surgery he has to do he does, as a rule, perfunctorily. How often, for example, does one see him open abscesses with a sublime disregard of the rules of asepsis, which he learned during his dressing days; how often disdain the minor details of obstetrical practice as if he were unconscious of the truth that the gynæcologist thrives upon the sins of omission of the obstetrician.

The time has passed when the practitioner—at least, in this country, where expert service is so easily at hand—has to deal with conditions demanding special aptitude, exceptional experience, and exquisite chirurgical dexterity. For that reason he will continue to send his patients to the hospital or the expert. The demand of a large private practice on the time and attention of the practitioner is far too great to permit of his gaining the necessary skill and manipulative experience in dealing with such operations as excisions, amputations, abdominal sections, and plastic methods. But it is not too great to permit him to pay a larger amount of attention to such operative procedures as legitimately fall within his scope. The perfection of local anæsthesia has brought such things as operations for defected septa of the nose, the removal of polypi and piles, the excision of superficial tumours, and the treatment of large ulcers by skin grafting easily within the reach of the practitioner. To perfect himself in these "minor operations," which are neither minor in the amount of care they demand nor in the amount of relief their successful performance brings to the patient, he should seize every opportunity to become proficient in the diagnosis of conditions which he is able to alleviate by such operative skill as he can attain to in the stress of private practice.

